# Protective Effectiveness of an Immunization Protocol Against the Toxic Effects of *Loxosceles intermedia* Venom in Rabbits

**DOI:** 10.3389/fvets.2022.852917

**Published:** 2022-05-31

**Authors:** Ana Luísa Soares de Miranda, Sabrina de Almeida Lima, Ana Flávia Machado Botelho, Marco Túlio Gomes Campos, Camila Eckstein, João Carlos Minozzo, Carlos Delfin Chávez-Olórtegui, Benito Soto-Blanco

**Affiliations:** ^1^Department of Veterinary Clinics and Surgery, Veterinary College, Federal University of Minas Gerais, Belo Horizonte, Brazil; ^2^Department of Biochemistry and Immunology, Institute of Biological Sciences, Federal University of Minas Gerais, Belo Horizonte, Brazil; ^3^Department of Veterinary Medicine, Veterinary College, Federal University of Goiás, Goiânia, Brazil; ^4^Department of Health of the State of Paraná, Production and Research Center of Immunobiologicals, Piraquara, Brazil

**Keywords:** brown spider, dermonecrosis, immunization, loxoscelism, spider bite

## Abstract

*Loxosceles* spp. (brown spiders) bites are responsible for the development of a syndrome consisting mainly of dermonecrotic lesions, and also systemic effects. Rabbits are one of the main experimental models used for better understanding the systemic and local effects of *Loxosceles* venom. The aim of this study is to evaluate the toxic and protective effects of rabbits immunized with *Loxosceles* spp. venom. Male New Zealand rabbits were allocated as a control group (CG; *n* = 5) that received adjuvant (Montanide) and phosphate-buffer saline (PBS), or as venom group (VG; *n* = 5) that received 21 μg of *Loxosceles* venom using Montanide as adjuvant. After five immunization cycles, a trial with 7 μg of *Loxosceles intermedia* (*L*. *intermedia*) venom was performed, and dermonecrotic lesions were measured. The rabbits were then euthanized, and their organs were collected for histopathology analysis. Rabbits that had undergone *Loxosceles* venom immunization protocol showed minor clinical disturbances during the experimental period. The used immunization protocol protected the rabbits against the toxic effect of the *Loxosceles* venom because they showed minor clinical disturbances during the experimental period.

## Introduction

The venom of *Loxosceles* spiders, popularly known as “brown spiders,” is a complex mixture of toxins enriched by low molecular mass peptides (1), which include phospholipases D, hyaluronidases, astacin-like metalloproteases, and venom allergens (2–7). Phospholipases D cleave tissue phospholipids, boosting the tissue injury and inflammation (8, 9). Hyaluronidases and astacin-like metalloproteases hydrolyze the components of the extracellular matrix (10, 11). The allergens are the translationally controlled tumor protein (TCTP) and the *Loxosceles* allergen-like toxin (LALLT) that stimulate the release of histamine (12, 13). The *Loxosceles* venom also contains numerous other toxins with toxic effects not entirely known (9).

*Loxosceles* bites are responsible for the development of a cutaneous syndrome consisting mainly of dermonecrotic lesions in at least 80% of cases. Bites are usually painless, and edema and erythema are formed within 2 to 6 h. After 24 to 36 h, the vasospasm and ischemia around the bite devolve into pale and red areas with pain. The lesion may spread by gravitation. Necrosis may occur days after the bite (2, 7, 14). Other effects of the venom include fever (2, 14), platelet aggregation (15) and hematological disturbances (16, 17), acute kidney injury (18), cardiotoxicity (19), and even brain damage (20). Pets, especially dogs (21–23) and cats (24), are sensible to the *Loxosceles* envenoming (21, 22).

The most effective treatment for the bite is the administration of the specific antivenom. The production of the antivenom involves the immunization of an animal, usually horses, to induce the production of specific neutralizing antibodies (7). However, little is known about the overall impacts on the health status of these animals, during and after venom contact, which could aid in better understanding venom dynamics. Therefore, the aim of this study is to evaluate the toxic and protective effects of rabbits immunized with *Loxosceles* spp. venom.

## Materials and Methods

### Rabbits, Venom, and Immunization Protocol

All procedures were conducted according to the animal welfare guidelines and the approval of the Ethical Committee for the Use of Animals of the Federal University of Minas Gerais (CEUA/UFMG), under protocol number 388/2017.

*Loxosceles* venom was obtained from spiders captured within Paraná and Santa Catarina states, Brazil. Specimens of *Loxosceles intermedia* (*L. intermedia*), *L. gaucho*, and *L. laeta* had their venom extracted after being restrained from feeding for 30 days and undergoing an electrical stimulus of 12 V applied on the cephalothorax region. The venom pool obtained was dehydrated and kept at −20°C, in the dark, until its use.

Ten male New Zealand rabbits (*Oryctolagus cuniculus*), weighing ~2.8 kg, were kept in individual cages, fed twice a day with commercial ration and water *ad libitum*, and closely monitored for any relevant clinical alteration. Five rabbits composed the control group (CG) and the other five rabbits, the venom group (VG). Immunization protocol and sampling moments are described in Table 1.

**Table 1:** Immunization protocol of rabbits from venom group (VG) using *Loxosceles* venom.

**Immunization** **status**	**Day of the** **cycle**	**Total venom** **amount**	**Venom amount per species of** ***L. intermedia, L. laeta*** **and** ***L. gaucho***	**PBS**	**Montanide**
T0	0	Clinical examination, blood sampling, ECG recordings			
T1	1	21 μg	7 μg	973.75 μL	1 mL
T2	15	21 μg	7 μg	973.75 μL	1 mL
T3	16	Clinical examination, blood sampling, ECG recordings			
T4	30	21 μg	7 μg	973.75 μL	1 mL
T5	45	21 μg	7 μg	973.75 μL	1 mL
T6	46	Clinical examination, blood sampling, ECG recordings			
T7	60	21 μg	7 μg	973.75 μL	1 mL
T8	62	Blood sampling, ECG recordings			
T9	68	Challenge with *L. intermedia* venom			
T10	75	Euthanasia			

The CG received 1 ml of Montanide (vaccine adjuvant) + 1 ml of phosphate-buffer saline (PBS), whereas the VG received 2.5 μg/kg (7 μg) of *L. intermedia*, 2.5 μg/kg of *L. laeta*, and 2.5 μg/kg of *L. gaucho* venoms diluted in 973.75 μl of PBS + 1 ml of Montanide. The final volume of both groups was 2.0 ml, which was injected subcutaneously in two different points under the dorsal surface of the skin near the nape of the neck, after local trichotomy. A total of five immunizations was performed. On day 68 of the immunization cycle, rabbits from both the groups were challenged by inoculation with 7 μg of *L. intermedia* venom on their ear. This amount was the minimum necrotizing dose, which was determined for the used venom using the protocol described by Furlanetto (25). Dermal lesions were measured 24, 48 and 72 h after the challenge using a ruler as well as ImageJ® software. To ensure control over the histopathological analysis of lesions caused by a high dose of venom, one rabbit in each group was not subjected to this challenge. On day 75, the rabbits were euthanized with 100 mg/kg of thiopental intravenously, according to guidelines established by the Brazilian Guide of Good Practices in Euthanasia (26).

### Hematological Evaluation

Blood samples were collected from the marginal ear vein before the first immunization and then after 16, 46, and 62 days. Vacuum tubes containing ethylenediaminetetraacetic acid (EDTA) and clot activator tubes were used for hematological and biochemical analyses, respectively. Hematological analyses were performed using an automated hematology analyzer (pocH-100Iv-Diff, Sysmex), and serum biochemistry was analyzed by an automatized biochemistry equipment (Cobas Mira Plus, Roche). Blood parameters evaluated were red blood cell count (RBC), packed cell volume (PCV), mean corpuscular volume (MCV), mean corpuscular hemoglobin (MCH), mean corpuscular hemoglobin concentration (MCHC), white blood cell count (WBC), red blood cell distribution width (RDW), lymphocytes, and sum of other WBC, such as neutrophils, monocytes, and basophils (OTH), total platelet count (PLT), mean platelet volume (MPV), platelet distribution width (PDW), platelet clump (P-LCR), urea, creatinine, alanine aminotransferase (ALT), aspartate transaminase (AST), alkaline phosphatase (ALP), gamma-glutamyl transferase (GGT), glucose, amylase, total proteins (TP), albumin, globulins, cholesterol, triglyceride, and lactate.

### Electrocardiography

The rabbits underwent an ECG evaluation using a portable 12-channel digital electrocardiograph (TEB ECG Vet', Tecnologia Eletrônica Brasileira) before immunization and then after 16, 46, and 62 days. ECG recordings were acquired in a quiet environment and with rabbits in lateral horizontal decubitus position. Recordings were made at 25 mm/sec speed and sensitivity of 1 cm = 1 mV. Bipolar (DI, DII, DIII) and augmented unipolar (aVR, aVL, aVF) leads were recorded. The following parameters were evaluated: cardiac rhythm; HR; P (ms); P (mV); PR, QRS, and QT intervals; R and T waves and ST segment levels. The cardiac axis was calculated according to Tilley (27).

### Histopathology

After euthanasia, an immediate necropsy was performed, and significant fragments of liver, kidney, spleen, heart, lungs, and skin were collected for microscopic evaluation. The fragments were fixated in 10% formaldehyde and afterward embedded in paraffin. Histological sections (4 μm thickness) of paraffin-embedded fragments were dyed with hematoxylin and eosin (HE) and periodic acid Schiff (P.A.S.) for pathological examination under light microscopy.

### ELISA

ELISA from rabbits' sera was performed in MaxiSorp plates purchased from NUNC. They were coated overnight at 4°C with 100 μl of a 5 μg/ml solution of *L. intermedia, L. gaucho*, and *L. laeta* venoms in 0.02 M sodium bicarbonate buffer, pH 9.6. After blocking (1% skimmed non-fat milk in PBS) and washing (0.05% Tween 20-PBS), sera from T0 and immune rabbits were added in serial dilution from 1/400 to 1/256,000 and incubated for 1 h at 37°C. The plates were washed and incubated with anti-rabbit IgG conjugated with horseradish peroxidase (HRP, Sigma-Aldrich A9292) diluted 1/4,000, for 1 h at 37°C. ELISA was carried out as described by Chavez-Olórtegui et al. (28). Absorbance values were determined at 492 nm using an ELISA plate reader (BIO-RAD, 680 models). Duplicate assays were taken for all samples, and means were calculated.

### Statistical Analysis

Statistical analysis was carried out using the SAS (version 9.0) software program. The obtained data were statistically analyzed using a mixed linear model approach of SAS (PROC MIXED), using first-order autocorrelation covariate structure. Animals were considered a random factor, with each nested within treatments, and measurements carried out repeatedly. The significance level was set at *p* < 0.05.

## Results and Discussion

The immunization protocol used in the present study was the injection of the venom of three brown spider species: *L. intermedia, L. laeta*, and *L. gaucho*. This protocol was found to promote the production of anti-loxoscelic venom polyvalent antibodies (29). The vaccine adjuvant was Montanide, a water-in-oil emulsion, mainly composed of mineral oil and a surfactant from the mannide monooleate family. Its mode of action is based on enhancing antigen-specific antibody titers and responses coming from cytotoxic T-lymphocyte. It was postulated that depot formation would slowly release antigens at the immunization site. There were however other mechanisms of action proposed, such as an inflammation promoter (thus stimulating the recruitment of antigen-presenting cells - APCs) and lymphocyte trapping (thereby enhancing lymphocyte accumulation in lymph nodes and optimizing contact with APCs) (30–32).

**Figure 1:**
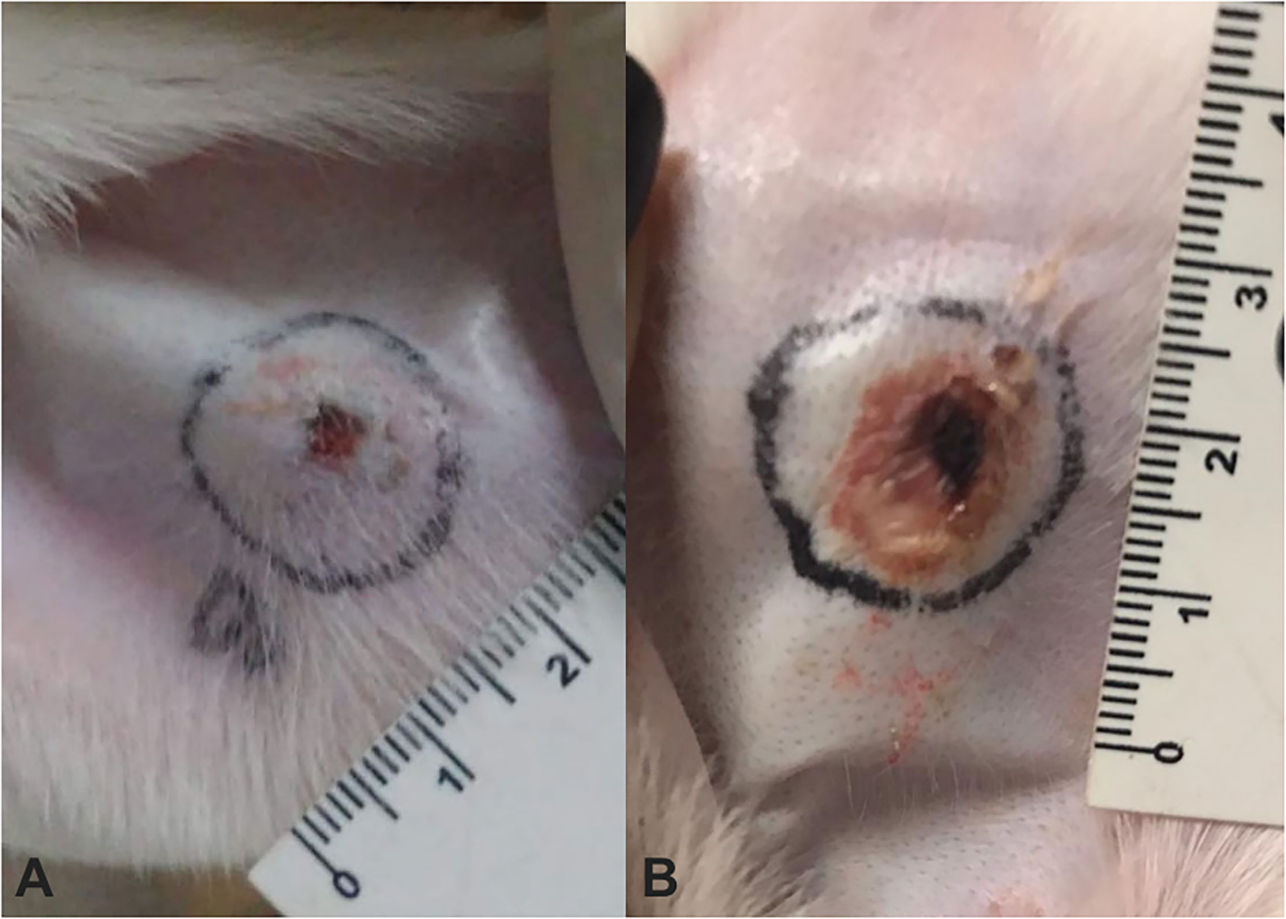
Measurement of dermonecrotic lesions after challenge using a 7 μg of *Loxosceles intermedia (L. intermedia)* venom in rabbits that have undergone immunization protocol using venom from *Loxosceles* spp. **(A)** Rabbit V1 from venom group (VG) showing a minimal lesion with absence of necrosis. **(B)** Rabbit C1 from control group (CG) showing a larger lesion area accompanied by necrosis.

**Figure 2:**
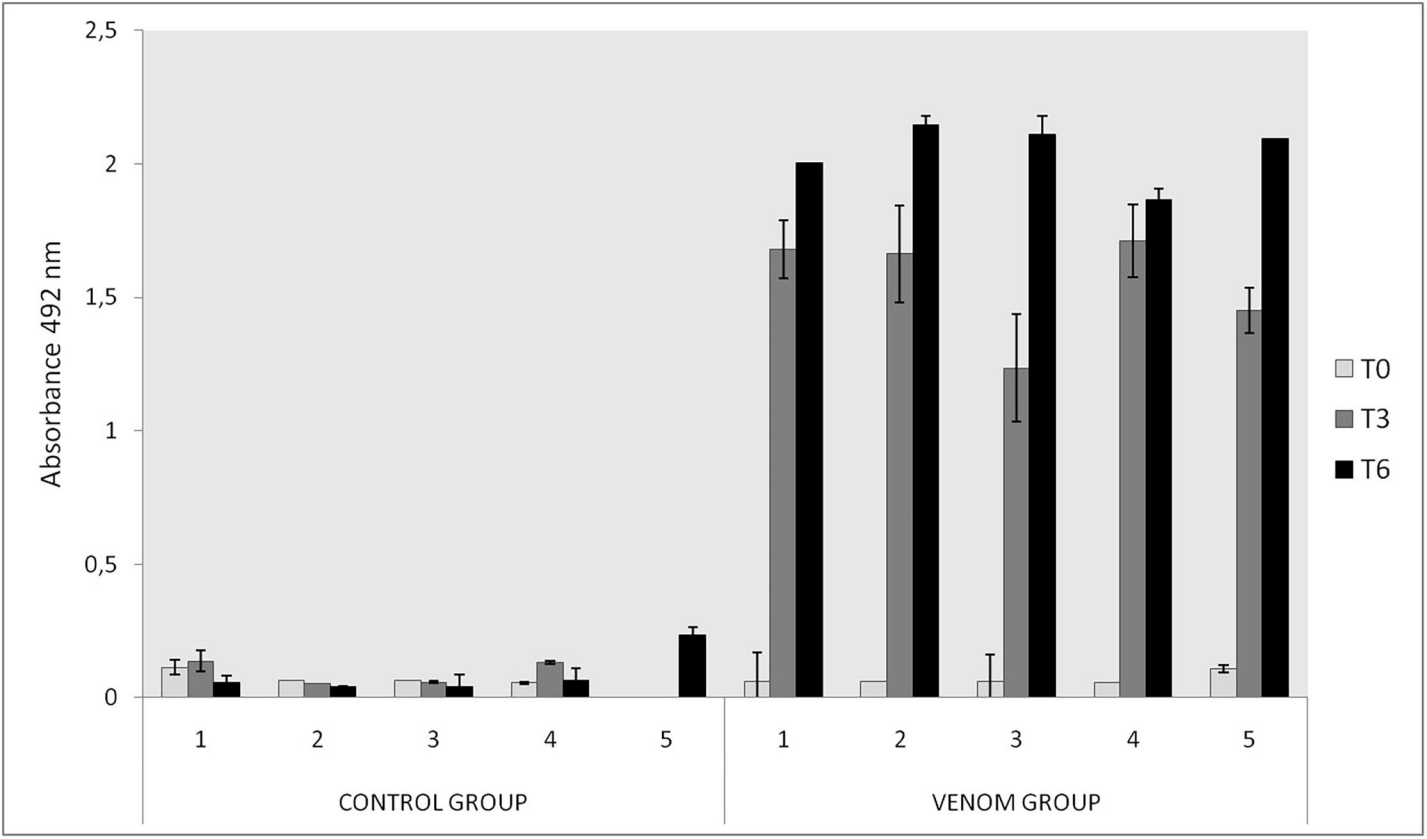
Immunoreactivity of rabbits immunized with *Loxosceles* spp. venom by ELISA in T0, T3, and T6. Plates were coated with a 5 μg/ml of each venom (*L. intermedia, L. gaucho*, and *L. laeta*) and sera was tested in 1/200 dilution and revealed with rabbit anti-IgG 1/5000 and o-phenylediamine dihydrochloride. The CG received Montanide and PBS and the VG received 21 μg of *Loxosceles* spp. venom and Montanide. The absorbance of samples was determined at 492 nm.

No relevant clinical change was found during immunizations, except a slight soreness on inoculation sites in some animals. After challenge with *L. intermedia* venom, necrotic lesions were observed on the rabbits' ear. Rabbits of the VG (Figure 1A) presented significant smaller dermal lesions (0.08 cm^2^ of lesion with 0.01 cm^2^ of necrosis) than those of the CG (1.08 cm^2^ of lesion with 0.11 cm^2^ of necrosis) (Figure 1B). Thus, an adequate sera conversion was achieved and neutralizing antibodies were successfully produced, as was also shown in ELISA assay (Figure 2).

Skin lesions diagnosed in the present study were characteristic of the dermonecrotic lesions previously described in loxoscelic envenomation (4, 33, 34). Rabbits of the CG presented more extensive lesions than those of the VG, probably because the immunization protocol was able to confer enough protective antibody titers. The pathophysiology of dermonecrosis is not yet fully elucidated. Phospholipase D is the main compound responsible for dermonecrosis, promoting neutrophilic infiltration, complement activation, platelet aggregation, edema, and increased vascular permeability (38, 39). A role for neutrophils in the inflammatory response is directly linked to endothelial dysfunction (33), which leads to indirect neutrophil activation, leading to up-regulation of interleukins (IL) 6 and 8, C-X-C motif chemokine ligands (CXCL) 1 and 2, and monocyte chemoattractant protein (MCP) 1 (40, 41). Endothelial dysfunction also plays an important role in dermonecrosis, since it occurs in both extracellular matrix and cellular surface, leading to subendothelial vacuoles and fibrin formation, accompanied by morphologic alterations, such as cellular retraction, reduction of intercellular adhesion, and disorganization of actin filaments. These disruptions on endothelial surfaces and cellular adhesion structures act directly on blood vessel stability and can cause leukocyte and platelet activation, increase in vascular permeability, and disseminated intravascular coagulation (42). As a result of endothelial dysfunction, platelets aggregate, which can cause dermonecrosis, since occluded dermal veins and arterioles cause hypoxia and degeneration of cells (43).

The results of the hematological examinations are presented in Table 2. WBC counts increased after T0 in both groups. This increase may be attributed to the adjuvant (Montanide) that increases the antigen uptake by APCs, activates or aids in maturing APCs (e.g., dendritic cells), besides inducing the production of immunoregulatory cytokines, activating inflammasomes, and inducing local inflammation and cellular recruitment (44). VG showed WBC counts above reference ranges for the species and significant different from CG in T3 and T8. This difference might be attributed to venom action. Phospholipase D, the main toxic compound of *Loxosceles* venom, is the primary inductor of both expression and secretion of inflammatory mediators, especially in fibroblasts. This induction culminates in the fibroblast secreting inflammatory mediators that contribute to monocyte recruitment, such as IL-6, IL-8, CXCL1/GRO-α, and CCL2/MCP-1 (41).

**Table 2:** Hematological examination of rabbits that underwent immunization protocols with *Loxosceles* venom + Montanide (VG) and rabbits that received Montanide + phosphate-buffer saline (PBS) [Control group (CG)].

**Parameter**	**Control group**	**Venom group**	**Reference values for rabbits (35)**
RBC (cell × 10^6^/μL) T0 T3 T6 T8	6.59 ± 0.17 ^a^ 6.31 ± 0.13 ^b^ 6.63 ± 0.15 ^a^ 6.61 ± 0.13 ^a^	6.66 ± 0.07 6.54 ± 0.08 6.62 ± 0.07 6.77 ± 0.02	5.4–7.6
			
PCV (%) T0 T3 T6 T8	42.6 ± 0.78 41.9 ± 0.86 42.9 ± 0.62 43.2 ± 0.94	42.2 ± 0.57 42.1 ± 0.51 42.2 ± 0.66 43.7 ± 1.11	33.0–50.0
WBC (cell × 10^3^/μL) T0 T3 T6 T8	8.25 ± 1.16 ^b^ 8.28 ± 1.13 ^b^ 12.1 ± 0.61 ^a^ 11.6 ± 1.32 ^a^	10.3 ± 1.37 ^c^ 13.5 ± 0.62 ^b,*^ 14.8 ± 0.32 ^a,b^ 1 16.7±± 1.16 ^a,*^	5.2–12.5
Lymphocytes (%) T0 T3 T6 T8	62.5 ± 4.45 60.6 ± 5.64 53.0 ± 2.94 53.0 ± 1.90	65.2 ± 3.30 ^a^ 45.9 ± 3.38 ^b,*^ 49.4 ± 3.55 ^b^ 47.2 ± 2.19 ^b^	30.0–85.0
OTHR (%)			
T0 T3 T6 T8	28.2 ± 3.35 32.5 ± 4.94 33.7 ± 3.33 36.3 ± 4.65	28.2 ± 2.70 ^b^ 46.0 ± 3.55 ^a,*^ 43.0 ± 2.25 ^a^ 44.2 ± 2.18 ^a^	
Eosinophils (%) T0 T3 T6 T8	17.5 ± 4.13 ^a^ 9.73 ± 2.89 ^b^ 18.3 ± 2.79 ^a^ 10.7 ± 3.06 ^a,b^	13.2 ± 4.14 8.12 ± 0.90 7.64 ± 1.98 * 8.65 ± 0.36	1.0–4.0
PLT (cell × 10^3^/μL) T0 T3 T6 T8	184.7 ± 37.8 ^b^ 147.0 ± 39.7 ^b^ 341.3 ± 41.1 ^a^ 263.7 ± 63.0 ^a,b^	277.8 ± 12.3 ^a,b^ 1 185.4±± 32.4 ^b^ 324.2 ± 24.4 ^a^ 251.3 ± 21.5 ^a,b^	250.0–650.0
PDW (fL) T0 T3 T6 T8	8.12 ± 0.55 ^b^ 9.17 ± 0.50 ^a^ 6.88 ± 0.32 ^c^ 6.40 ± 0.30 ^c^	8.10 ± 0.39 ^b^ 9.78 ± 0.62 ^a^ 7.44 ± 0.40 ^b^ 7.05 ± 0.37 ^b^	
MPV (fL) T0 T3 T6 T8	7.94 ± 0.32 ^b^ 8.68 ± 0.29 ^a^ 7.15 ± 0.26 ^c^ 6.83 ± 0.15 ^c^	8.03 ± 0.28 ^b^ 8.94 ± 0.33 ^a^ 7.52 ± 0.23 ^b,c^ 7 7.35±± 0.30 ^c^	
P-LCR (%) T0 T3 T6 T8	9.00 ± 2.01 ^b^ 14.3 ± 2.55 ^a^ 5.23 ± 1.05 ^b^ 4.53 ± 0.54 ^b^	9.23 ± 1.91 ^b^ 14.7 ± 2.80 ^a^ 6.66 ± 0.95 ^b^ 5.93 ± 1.09 ^b^	

*RBC, red blood cell count; PCV, packed cell volume; WBC, white blood cell count; OTHR, sum of other WBC (neutrophils, monocytes, and basophils); PLT, platelets; PDW, platelet distribution width; MPV, mean platelet volume; P-LCR, platelet clump*.

*^a, b, c^ Different letters in the same column show statistical difference between experimental moments in the same group (mixed linear model, p < 0.05)*.

* *Statistical difference to control group in the same day (mixed linear model, p < 0.05)*.

*Data are presented as mean ± SEM*.

A decrease in lymphocytes, accompanied by an increase in neutrophils and monocytes, was observed in both groups during all sampling periods and were statistically significant between the VG and CG, with the VG presenting higher values. It is likely that this finding is due to the venom, since neutrophils are largely recruited in acute inflammation (45) and Montanide itself does not affect the number of circulating leukocytes (46). An interesting finding, however, was regarding eosinophils. Both groups kept eosinophil values way above the reference values for the species. *Loxosceles* venom has a TCTP toxin, with a histaminergic effect related to pro-inflammatory properties, acting as an allergen (12). Allergic reactions are accounted for eosinophilia, which might explain the results from the VG but not from the CG. A plausible explanation is that rabbits' neutrophils have eosinophilic granules, which can make them be misclassified as eosinophils (35). Thus, a more pronounced neutrophilia could be happening in both the groups and was underestimated due to the eosinophil overestimated count. However, eosinophilia was observed in histopathology analysis of some organs.

The results of the biochemical panel are presented at Table 3. Glucose levels showed statistical significance between the VG and CG in T3 and T6. Increased glucose could be related to stress and pain (47). In fact, rabbits in the present study needed to be handled for sampling, which could have caused stress, and manipulation could trigger a pain response in the already sore injection sites. However, it is plausible to attribute the variations to chance since glucose levels were within normal ranges.

**Table 3:** Biochemical panel of rabbits that underwent immunization protocols with *Loxosceles* venom + Montanide (VG) and rabbits that received Montanide + PBS (CG).

**Parameter**	**Control group**	**Venom group**	**Reference values for rabbits (36)**
BUN (mg/dL) T0 T3 T6	20.6 ± 0.47 16.7 ± 1.28 20.0 ± 4.28	19.8 ± 0.84 15.8 ± 1.27 15.8 ± 1.33	10–30
Creatinine (mg/dL) T0 T3 T6	1.52 ± 0.06 1.63 ± 0.25 1.51 ± 0.14	1.64 ± 0.10 1.42 ± 0.21 1.43 ± 0.13	0.5–2.5
ALT (U/L) T0 T3 T6	33.0 ± 6.92 ^a^ 29.2 ± 5.18 ^a^ 13.4 ± 1.03 ^b^	27.7 ± 3.32 ^a^ 15.0 ± 1.60 ^b,*^ 13.8 ± 2.70 ^b^	25.0–65.0
AST (U/L) T0 T3 T6	31.6 ± 6.35 27.0 ± 4.62 17.0 ± 1.80	27.2 ± 3.82 18.3 ± 5.87 21.3 ± 4.12	20.0–120.0
ALP (U/L) T0 T3 T6	56.2 ± 4.89 48.7 ± 5.54 33.0 ± 8.58	83.9 ± 17.0 63.0 ± 10.6 51.8 ± 10.7	10.0–86.0
GGT (U/L) T0 T3 T6	14.3 ± 2.55 ^b^ 40.2 ± 7.70 ^a^ 16.4 ± 1.21 ^b^	13.4 ± 1.85 14.3 ± 3.55 * 16.8 ± 1.90	10.0–98.0
Glucose (mg/dL) T0 T3 T6	94.2 ± 3.09 ^a^ 60.7 ± 9.03 ^b^ 105.2 ± 4.03 ^a^	103.3 ± 6.23 ^b^ 102.7 ± 10.2 ^b,*^ 139.0 ± 18.2 ^a,*^	74.0–148.0
Amylase (U/L) T0 T3 T6	409.0 ± 28.2 328.8 ± 20.0 322.7 ± 73.4	329.4 ± 86.7 347.5 ± 25.3 295.0 ± 22.6	200.0–500.0
TP (g/dL) T0 T3 T6	6.10 ± 0.30 6.09 ± 0.19 5.80 ± 0.08	6.87 ± 0.14 ^a,*^ 5.93 ± 0.20 ^b^ 6.04 ± 0.21 ^b^	5.0–7.5
Albumin (g/dL) T0 T3 T6	3.61 ± 0.40 ^a,b^ 2 2.91±± 0.24 ^b^ 3.76 ± 0.09 ^a^	3.88 ± 0.15 3.75 ± 0.11 * 3.80 ± 0.19	2.7–5.0
Globulins (g/dL) T0 T3 T6	2.49 ± 0.11 ^b^ 3.18 ± 0.13 ^a^ 2.04 ± 0.17 ^c^	2.98 ± 0.10 ^a,*^ 2.17 ± 0.25 ^b,*^ 2.24 ± 0.09 ^a^	1.5–2.7
Cholesterol (mg/dL) T0 T3 T6	31.2 ± 2.29 42.5 ± 3.85 42.3 ± 5.43	31.2 ± 3.18 30.0 ± 3.82 * 32.4 ± 3.34 *	10.0–100.0
Triglycerides (mg/dL) T0 T3 T6	73.1 ± 5.47 71.1 ± 13.3 79.5 ± 27.16	75.5 ± 8.77 101.1 ± 24.3 72.7 ± 8.45	50.0–200.0
Lactate (mol/L) T0 T3 T6	9.81 ± 2.18 10.2 ± 2.57 5.81 ± 0.45	11.7 ± 2.77 6.00 ± 0.97 7.61 ± 1.00	8.11–21.2

*BUN, blood urea nitrogen; ALT, alanine aminotransferase; AST, aspartate transaminase; ALP, alkaline phosphatase; GGT, gamma-glutamyl transferase; TP, total protein*.

*^a, b, c^ Different letters in the same column show statistical difference between experimental moments in the same group (mixed linear model, p < 0.05)*.

* *Statistical difference to control group in the same day (mixed linear model, p < 0.05)*.

*Data are presented as mean ± SEM*.

**Figure 3:**
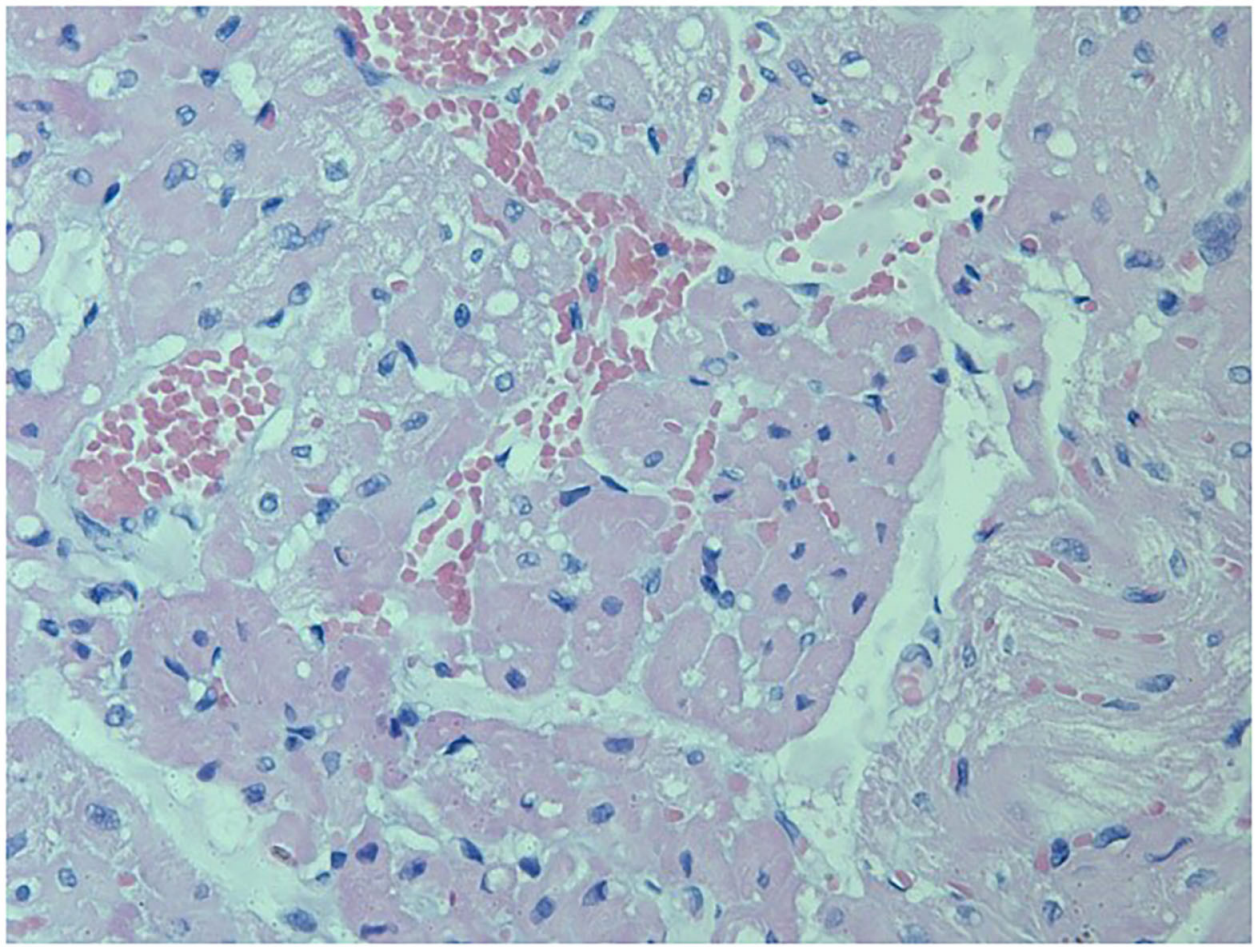
Left ventricle from a rabbit from the VG showing severe and diffuse congestion, with multifocal hemorrhage areas. Multifocal cardiomyocyte degeneration was also observed. Besides undergoing immunization protocols, this rabbit also underwent the trial period receiving a 7μg of *L. intermedia* venom (HE, 400X). Periodic acid Schiff (P.A.S.) stain was not positive.

**Figure 4:**
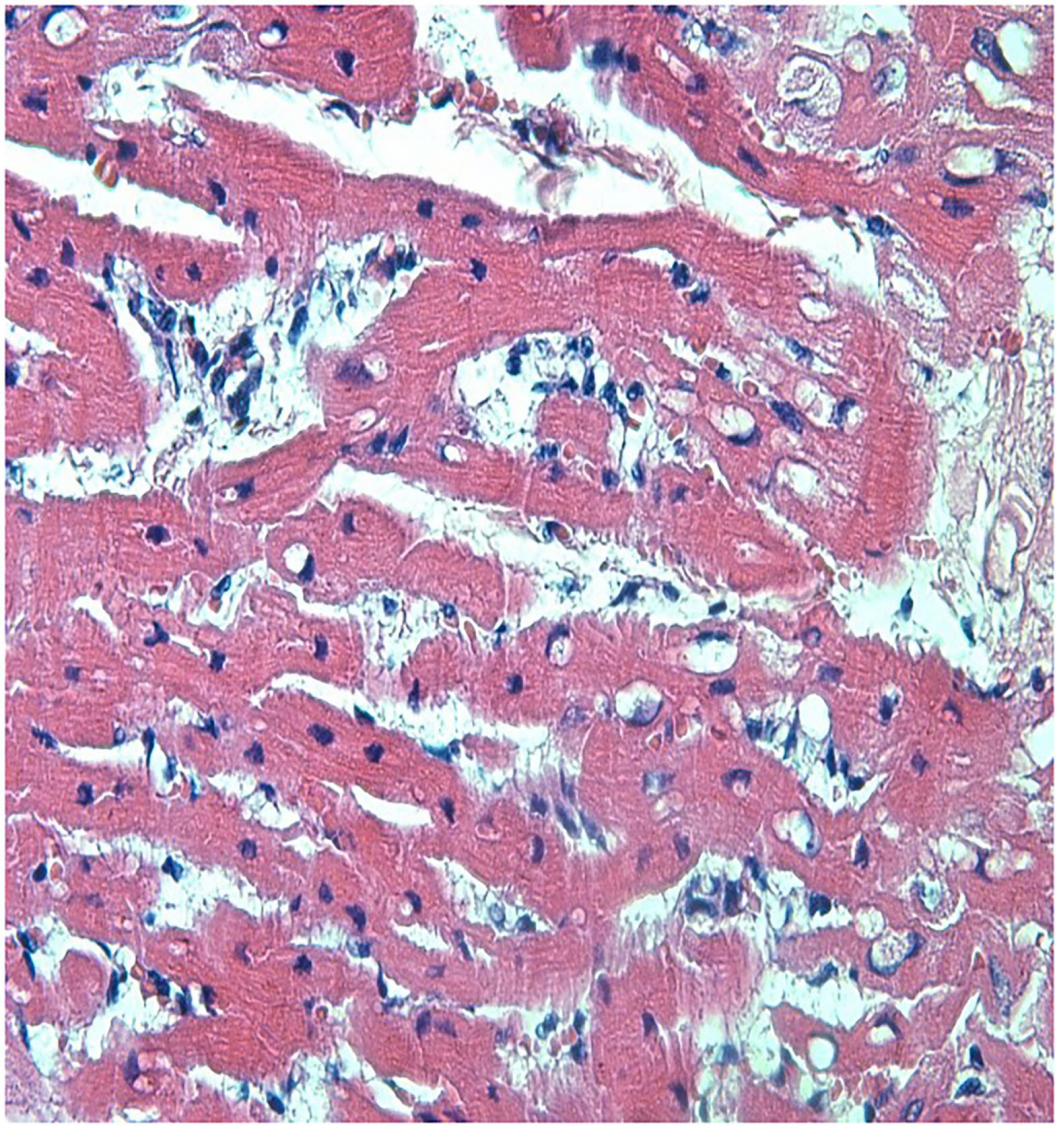
Left ventricle from a rabbit from the VG showing severe and diffuse congestion, with lymph-histiocytic infiltrate near blood vessels. Discrete multifocal cardiomyocyte degeneration was also observed. This rabbit did not partake the trial period and was inoculated with venom only accompanied by adjuvant (HE, 400X). P.A.S. stain was not positive.

*Loxosceles intermedia* venom may promote cardiotoxic effects (19). ECG recordings made on four occasions revealed that all the rabbits kept the ECG variables within reference ranges, and no significant difference was observed between the groups (Table 4). On the other hand, the histological analysis of heart revealed lesions in both groups (Figures 3, 4). The CG showed mild cardiomyocyte degeneration, which suggests that the adjuvant (Montanide) may be the cause, but this was not previously reported in the literature. Cardiomyocyte degeneration, in a more severe degree, was observed in the VG and rabbits in the CG that had undergone challenge with *L. intermedia* venom. VG rabbits also presented lymph-histiocytic infiltrate and hemorrhagic areas. *L. intermedia* venom promoted impairment of cardiac function mainly due to disruptions in calcium flow and abnormal increase of its intracellular concentration (19). Rats injected with *Loxosceles apachea* venom also developed hemorrhages that were attributed to degradation of laminin γ (20).

**Table 4:** ECG recordings of rabbits that underwent immunization protocols with *Loxosceles* venom + Montanide (VG) and rabbits that received Montanide + PBS (CG).

**Parameter**	**Control group**	**Venom group**	**Reference values for rabbits (37)**
Heart rate (bpm) T0 T3 T6 T8	196.2 ± 13.11 ^a,b^ 199.0± 8.42 ^b^ 179.3 ± 6.63 ^a^ 192.9 ± 9.75 ^a,b^	226.3 ± 8.86 ^a^ 210.5 ± 10.85 ^b^ 210.4 ± 18.95 ^b^ 220.4 ± 18.99 ^a,b^	198.0–330.0
P (ms) T0 T3 T6 T8	35.3 ± 2.64 ^b^ 38.3 ± 2.49 ^a,b^ 3 37.9± 2.22 ^a,b^ 4 47.2± 7.33 ^a^	39.4 ± 2.95 39.2 ± 3.10 41.8 ± 2.51 44.8 ± 1.77	10.0–50.0
P (mV) T0 T3 T6 T8	0.04 ± 0 ^b^ 0.05 ± 0.01 ^a,b^ 0 0.03± 0 ^b^ 0.06 ± 0.01 ^a^	0.03 ± 0.01 0.04 ± 0.01 0.04 ± 0.01 0.05 ± 0	0.04–0.12
PR (ms) T0 T3 T6 T8	55.2 ± 9.09 57.5 ± 8.07 69.6 ± 8.06 68.3 ± 7.68	67.4 ± 9.58 56.5 ± 7.41 66.0 ± 5.32 75.1 ± 1.94	40.0–80.0
QRS (ms) T0 T3 T6 T8	47.4 ± 4.34 40.3 ± 2.14 46.3 ± 4.41 40.6 ± 3.56	46.5 ± 2.88 43.3 ± 4.46 44.0 ± 4.25 45.7 ± 3.02	20.0–60.0
R (mV) T0 T3 T6 T8	0.12 ± 0.07 0.11 ± 0.08 0.04 ± 0.06 0.06 ± 0.11	0.14 ± 0.05 0.15 ± 0.02 0.11 ± 0.01 0.12 ± 0.03	0.03–0.39
QT (ms) T0 T3 T6 T8	131.6 ± 14.5 ^b^ 165.8 ± 13.5 ^a^ 142.2 ± 8.10 ^a,b^ 145.8± 2.25 ^a,b^	127.8 ± 6.79 139.5 ± 10.5 122.6 ± 17.9 142.1 ± 9.89	80.0–160.0
T (mV) T0 T3 T6 T8	0.16 ± 0.04 0.11 ± 0.03 0.07 ± 0.01 0.06 ± 0.01	0.12 ± 0.08 0.05 ± 0.02 0.06 ± 0.02 0.08 ± 0.02	0.05–0.17

*^a, b, c^ Different letters in the same column show statistical difference between experimental moments in the same group (mixed linear model, p < 0.05).*

*Data are presented as mean ± SEM*.

Even though no change was found in the serum levels of urea and creatinine, kidney damage was observed in the histopathology analysis of both groups. The CG rabbits showed mild tubular cell degeneration, which may be due to Montanide, but this has not been reported previously. The VG animals presented fibrosis and moderate lymph-histiocytic infiltrate, inferring a slight renal impairment. In the same way, rats showed a sudden and significant decline in glomerular filtration rate, renal blood flow, and urinary output associated to an increase in renal vascular resistance after intravenous injection of 240 μg/kg *L. gaucho* venom (18). These changes resulted in acute kidney epithelial tubular, which may be attributed to the high dose of venom and the intravenous administration.

*Loxosceles intermedia* venom holds a direct hepatotoxic effect due to direct venom action in degenerating hepatocytes membrane and neutrophil infiltration (48). In the present study, the pathological examination of the liver revealed glycogenic degeneration in VG rabbits. Glycogenic degeneration could be due to a decrease in glycogen mobilization, and its accumulation could cause steatosis that can evolve into fibrosis. These histopathological alterations in rabbits from the present study were not accompanied by increased serum activities of liver-related enzymes (GGT, ALT, AST, and ALP), allowing to classify these alterations as mild.

In summary, the used immunization protocol-protected rabbits against the toxic effect of the *Loxosceles* venom because they showed minor clinical disturbances during the experimental period. These findings were corroborated by the lack of ECG alterations and the minor histopathological alterations observed in key-organs, such as kidneys and liver.

## Data Availability Statement

The raw data supporting the conclusions of this article will be made available by the authors, without undue reservation.

## Ethics Statement

The animal study was reviewed and approved by Ethical Committee for the Use of Animals of the Federal University of Minas Gerais (CEUA/UFMG), under protocol number 388/2017.

## Author Contributions

AM, SL, MG, and JM performed laboratory examinations. AM and AB performed clinical and electrocardiographic examinations. CE performed and interpreted the pathological examinations. CC-O and BS-B designed the study. AM, CC-O, and BS-B drafted the manuscript. All authors critically revised the manuscript and gave final approval.

## Funding

This study was partially supported by the Conselho Nacional de Desenvolvimento Científico e Tecnológico–Brazil (CNPQ) (Process 311182/2017-8).

## Conflict of Interest

The authors declare that the research was conducted in the absence of any commercial or financial relationships that could be construed as a potential conflict of interest.

## Publisher's Note

All claims expressed in this article are solely those of the authors and do not necessarily represent those of their affiliated organizations, or those of the publisher, the editors and the reviewers. Any product that may be evaluated in this article, or claim that may be made by its manufacturer, is not guaranteed or endorsed by the publisher.
